# Benzyl *N*′-(2-chloro­benzyl­idene)hydrazinecarbodithio­ate

**DOI:** 10.1107/S1600536808034892

**Published:** 2008-11-08

**Authors:** Zhi-Qiang Shi, Ning-Ning Ji, Qin-Qin Ji

**Affiliations:** a Department of Materials Science and Chemical Engineering, Taishan University, 271021 Taian, Shandong, People’s Republic of China; b Department of Chemistry, Taishan University, 271021 Taian, Shandong, People’s Republic of China; c Department of Chemical Engineering and Technology, School of Chemical Engineering and Technology, China University of Mining and Technology, 221116 Xuzhou, Jiangsu, People’s Republic of China

## Abstract

The asymmetric unit of the title compound, C_15_H_13_ClN_2_S_2_, contains two independent mol­ecules, which are linked into a pseudo-centrosymmetric dimer by inter­molecular N—H⋯S hydrogen bonds. The aromatic rings form dihedral angles of 67.06 (3) and 81.85 (2)° in the two independent mol­ecules.

## Related literature

For the biomedical properties of ligands derived from *S*-benzyl­dithio­carbaza­te, see: Ali *et al.* (2001 ▶, 2002 ▶); Tarafder *et al.* (2001 ▶, 2008 ▶). For bond-length data, see: Allen *et al.* (1987 ▶).
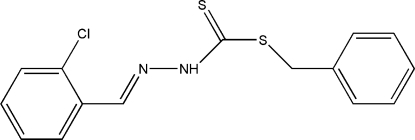


## Experimental

### 

#### Crystal data

C_15_H_13_ClN_2_S_2_
*M* *_r_* = 320.84
Triclinic, 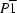

*a* = 11.877 (2) Å
*b* = 11.906 (2) Å
*c* = 12.623 (3) Å
α = 68.242 (3)°
β = 71.116 (4)°
γ = 82.335 (4)°
*V* = 1568.4 (5) Å^3^
*Z* = 4
Mo *K*α radiation
μ = 0.50 mm^−1^
*T* = 295 (2) K
0.12 × 0.10 × 0.06 mm

#### Data collection

Bruker APEXII CCD area-detector diffractometer
Absorption correction: multi-scan (*SADABS*; Bruker, 2005 ▶) *T*_min_ = 0.942, *T*_max_ = 0.971
8397 measured reflections
5524 independent reflections
3436 reflections with *I* > 2σ(*I*)
*R*_int_ = 0.026

#### Refinement

*R*[*F* ^2^ > 2σ(*F* ^2^)] = 0.046
*wR*(*F* ^2^) = 0.127
*S* = 0.97
5524 reflections
361 parameters
H-atom parameters constrained
Δρ_max_ = 0.44 e Å^−3^
Δρ_min_ = −0.46 e Å^−3^

### 

Data collection: *APEX2* (Bruker, 2005 ▶); cell refinement: *APEX2* and *SAINT* (Bruker, 2005 ▶); data reduction: *SAINT*; program(s) used to solve structure: *SHELXTL* (Sheldrick, 2008 ▶); program(s) used to refine structure: *SHELXTL*; molecular graphics: *SHELXTL*; software used to prepare material for publication: *SHELXTL* .

## Supplementary Material

Crystal structure: contains datablocks global, I. DOI: 10.1107/S1600536808034892/cv2453sup1.cif

Structure factors: contains datablocks I. DOI: 10.1107/S1600536808034892/cv2453Isup2.hkl

Additional supplementary materials:  crystallographic information; 3D view; checkCIF report

## Figures and Tables

**Table 1:** Hydrogen-bond geometry (Å, °)

*D*—H⋯*A*	*D*—H	H⋯*A*	*D*⋯*A*	*D*—H⋯*A*
N1—H1⋯S4	0.86	2.56	3.405 (3)	166
N3—H3*A*⋯S2	0.86	2.60	3.451 (3)	169

**Fig. 1.**
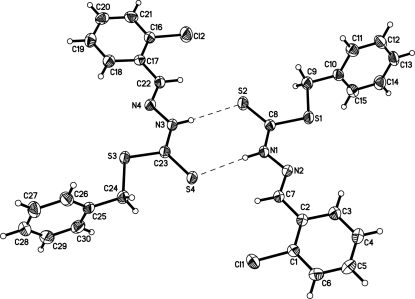
The molecular structure of (I), with displacement ellipsoids drawn at the 30% probability level. Dashed lines indicate hydrogen bonds.

## supplementary crystallographic information

### Comment

In recent years, the intriguing coordination chemistry and increasingly important biomedical properties of ligands derived from *S*-benzyldithiocarbazate(SBDTC) have received much attention (Ali *et al.*, 2001, 2002; Tarafder *et al.*, 2001, 2008). In order to search for new ligands derived from SBDTC, the title compound, (I), was synthesized. Herewith we present its crystal structure.

In (I), all bond lengths and angles are normal (Allen *et al.*, 1987). The C=N bond length in the independent molecules are 1.279 (3) Å(C7=N2) and 1.271 (4) Å(C22=N4), respectively, showing the double-bond character. The C=S bond lengths of 1.656 (3) Å(S2=C8) and 1.661 (3) Å(S4=C23) are intermediate between the values of 1.82Å for a C—S single bond and 1.56Å for a C=S double bond. The C=N—N angles in the independent molecule of 115.5 (2)° and 115.6 (3)° are significantly smaller than the ideal value of 120° expected for *sp*^2^-hybridized N atoms. This is probably a consequence of repulsion between the nitrogen lone pairs and the adjacent N bonds.

Two independent molecules are linked by N—H···S hydrogen bonds (Table 1) into pseudo-centrosymmetric dimers (Fig. 1).

### Experimental

The title compound was synthesized by the reaction of hydrazinecarbodithioic acid benzyl ester(1 mmol, 198.3 mg) with 2-chloro-benzaldehyde(1 mmol, 140.6 mg) in ethanol(20 ml) under reflux conditions (343 K) for 6 h. The solvent was removed and the solid product recrystallized from tetrahydrofuran. After five days yellow crystals suitable for X-ray diffraction study were obtained.

### Refinement

All H atoms were placed in idealized positions (C—H = 0.93— 0.97 Å, N—H = 0.86 Å) and refined as riding atoms. For those bound to C, *U*_iso_(H) = 1.2 or 1.5*U*_eq_(C). while for those bound to N, *U*_iso_(H) = 1.2 *U*_eq_(N).

### Crystal data

**Table:**

C_15_H_13_ClN_2_S_2_	*Z* = 4
*M**_r_* = 320.84	*F*_000_ = 664
Triclinic, *P*1	*D*_x_ = 1.359 Mg m^−^^3^
Hall symbol: -P 1	Mo *K*α radiation λ = 0.71073 Å
*a* = 11.877 (2) Å	Cell parameters from 1618 reflections
*b* = 11.906 (2) Å	θ = 2.6–24.6º
*c* = 12.623 (3) Å	µ = 0.50 mm^−^^1^
α = 68.242 (3)º	*T* = 295 (2) K
β = 71.116 (4)º	Block, yellow
γ = 82.335 (4)º	0.12 × 0.10 × 0.06 mm
*V* = 1568.4 (5) Å^3^	

### Data collection

**Table:**

Bruker APEXII CCD area-detector diffractometer	5524 independent reflections
Radiation source: fine-focus sealed tube	3436 reflections with *I* > 2σ(*I*)
Monochromator: graphite	*R*_int_ = 0.026
*T* = 295(2) K	θ_max_ = 25.1º
φ and ω scans	θ_min_ = 1.8º
Absorption correction: multi-scan(SADABS; Bruker, 2005)	*h* = −11→14
*T*_min_ = 0.942, *T*_max_ = 0.971	*k* = −14→13
8397 measured reflections	*l* = −14→14

### Refinement

**Table:**

Refinement on *F*^2^	Secondary atom site location: difference Fourier map
Least-squares matrix: full	Hydrogen site location: inferred from neighbouring sites
*R*[*F*^2^ > 2σ(*F*^2^)] = 0.046	H-atom parameters constrained
*wR*(*F*^2^) = 0.127	*w* = 1/[σ^2^(*F*_o_^2^) + (0.0533*P*)^2^ + 0.4623*P*] where *P* = (*F*_o_^2^ + 2*F*_c_^2^)/3
*S* = 0.97	(Δ/σ)_max_ = 0.001
5524 reflections	Δρ_max_ = 0.44 e Å^−^^3^
361 parameters	Δρ_min_ = −0.46 e Å^−^^3^
Primary atom site location: structure-invariant direct methods	Extinction correction: none

### Special details

**Table:**

Geometry. All e.s.d.'s (except the e.s.d. in the dihedral angle between two l.s. planes) are estimated using the full covariance matrix. The cell e.s.d.'s are taken into account individually in the estimation of e.s.d.'s in distances, angles and torsion angles; correlations between e.s.d.'s in cell parameters are only used when they are defined by crystal symmetry. An approximate (isotropic) treatment of cell e.s.d.'s is used for estimating e.s.d.'s involving l.s. planes.
Refinement. Refinement of *F*^2^ against ALL reflections. The weighted *R*-factor *wR* and goodness of fit *S* are based on *F*^2^, conventional *R*-factors *R* are based on *F*, with *F* set to zero for negative *F*^2^. The threshold expression of *F*^2^ > σ(*F*^2^) is used only for calculating *R*-factors(gt) *etc*. and is not relevant to the choice of reflections for refinement. *R*-factors based on *F*^2^ are statistically about twice as large as those based on *F*, and *R*- factors based on ALL data will be even larger.

### Fractional atomic coordinates and isotropic or equivalent isotropic displacement parameters (Å^2^)

**Table:**

	*x*	*y*	*z*	*U*_iso_*/*U*_eq_	
Cl2	0.61916 (10)	0.15814 (11)	0.57081 (9)	0.0994 (4)	
S3	0.36389 (7)	0.66976 (8)	0.21058 (7)	0.0541 (3)	
S4	0.15028 (7)	0.61729 (8)	0.43141 (7)	0.0566 (3)	
N3	0.3563 (2)	0.5043 (2)	0.4167 (2)	0.0514 (7)	
H3A	0.3270	0.4634	0.4914	0.062*	
N4	0.4704 (2)	0.4789 (2)	0.3570 (2)	0.0478 (7)	
C16	0.6959 (3)	0.2483 (3)	0.4266 (3)	0.0544 (9)	
C17	0.6419 (3)	0.3506 (3)	0.3648 (3)	0.0455 (8)	
C18	0.7096 (3)	0.4188 (3)	0.2498 (3)	0.0554 (9)	
H18	0.6770	0.4888	0.2056	0.066*	
C19	0.8242 (3)	0.3835 (3)	0.2014 (3)	0.0606 (10)	
H19	0.8678	0.4290	0.1244	0.073*	
C20	0.8742 (3)	0.2812 (4)	0.2663 (3)	0.0617 (10)	
H20	0.9518	0.2584	0.2332	0.074*	
C21	0.8112 (3)	0.2133 (3)	0.3785 (3)	0.0631 (10)	
H21	0.8452	0.1443	0.4224	0.076*	
C22	0.5211 (3)	0.3882 (3)	0.4168 (3)	0.0504 (8)	
H22	0.4802	0.3449	0.4955	0.061*	
C23	0.2902 (3)	0.5914 (3)	0.3608 (3)	0.0441 (8)	
C24	0.2502 (3)	0.7786 (3)	0.1668 (3)	0.0612 (10)	
H24A	0.1772	0.7372	0.1861	0.073*	
H24B	0.2335	0.8347	0.2097	0.073*	
C25	0.2944 (3)	0.8461 (3)	0.0345 (3)	0.0480 (8)	
C26	0.3717 (3)	0.9404 (3)	−0.0123 (3)	0.0609 (10)	
H26	0.3981	0.9628	0.0389	0.073*	
C27	0.4111 (3)	1.0026 (3)	−0.1335 (3)	0.0689 (11)	
H27	0.4636	1.0661	−0.1634	0.083*	
C28	0.3730 (3)	0.9712 (4)	−0.2098 (3)	0.0684 (11)	
H28	0.3994	1.0132	−0.2916	0.082*	
C29	0.2967 (3)	0.8785 (4)	−0.1655 (3)	0.0716 (11)	
H29	0.2702	0.8573	−0.2172	0.086*	
C30	0.2579 (3)	0.8152 (3)	−0.0442 (3)	0.0623 (10)	
H30	0.2065	0.7509	−0.0151	0.075*	
Cl1	−0.22425 (9)	0.74778 (9)	0.55632 (8)	0.0702 (3)	
S1	0.06032 (8)	0.28590 (8)	0.93397 (7)	0.0589 (3)	
S2	0.25595 (8)	0.30266 (9)	0.70505 (7)	0.0619 (3)	
N1	0.0603 (2)	0.4336 (2)	0.7232 (2)	0.0497 (7)	
H1	0.0857	0.4679	0.6469	0.060*	
N2	−0.0472 (2)	0.4698 (2)	0.7859 (2)	0.0466 (7)	
C1	−0.2804 (3)	0.6881 (3)	0.7111 (3)	0.0492 (8)	
C2	−0.2159 (3)	0.6018 (3)	0.7786 (3)	0.0444 (8)	
C3	−0.2674 (3)	0.5582 (3)	0.9025 (3)	0.0577 (9)	
H3	−0.2269	0.5002	0.9504	0.069*	
C4	−0.3772 (3)	0.5999 (4)	0.9548 (3)	0.0728 (11)	
H4	−0.4098	0.5708	1.0375	0.087*	
C5	−0.4383 (3)	0.6844 (4)	0.8849 (4)	0.0804 (13)	
H5	−0.5128	0.7118	0.9205	0.097*	
C6	−0.3909 (3)	0.7286 (4)	0.7634 (3)	0.0669 (10)	
H6	−0.4329	0.7857	0.7164	0.080*	
C7	−0.1006 (3)	0.5565 (3)	0.7233 (3)	0.0461 (8)	
H7	−0.0652	0.5910	0.6413	0.055*	
C8	0.1257 (3)	0.3461 (3)	0.7791 (3)	0.0446 (8)	
C9	0.1668 (3)	0.1677 (3)	0.9769 (3)	0.0613 (10)	
H9A	0.1802	0.1150	0.9308	0.074*	
H9B	0.2421	0.2030	0.9614	0.074*	
C10	0.1176 (3)	0.0961 (3)	1.1086 (3)	0.0476 (8)	
C11	0.1692 (3)	0.1043 (3)	1.1888 (3)	0.0572 (9)	
H11	0.2325	0.1561	1.1620	0.069*	
C12	0.1273 (3)	0.0359 (3)	1.3090 (3)	0.0639 (10)	
H12	0.1632	0.0415	1.3622	0.077*	
C13	0.0338 (3)	−0.0398 (3)	1.3501 (3)	0.0632 (10)	
H13	0.0060	−0.0854	1.4310	0.076*	
C14	−0.0190 (3)	−0.0484 (3)	1.2711 (3)	0.0672 (11)	
H14	−0.0827	−0.0999	1.2983	0.081*	
C15	0.0231 (3)	0.0200 (3)	1.1512 (3)	0.0617 (10)	
H15	−0.0132	0.0144	1.0982	0.074*	

### Atomic displacement parameters (Å^2^)

**Table:**

	*U*^11^	*U*^22^	*U*^33^	*U*^12^	*U*^13^	*U*^23^
Cl2	0.0801 (8)	0.0990 (9)	0.0654 (7)	0.0195 (6)	−0.0101 (6)	0.0142 (6)
S3	0.0454 (5)	0.0542 (6)	0.0410 (5)	0.0071 (4)	−0.0009 (4)	−0.0054 (4)
S4	0.0451 (5)	0.0626 (6)	0.0416 (5)	0.0100 (4)	−0.0017 (4)	−0.0085 (4)
N3	0.0410 (16)	0.0570 (18)	0.0386 (15)	0.0077 (13)	−0.0044 (12)	−0.0063 (13)
N4	0.0396 (15)	0.0508 (17)	0.0441 (16)	0.0053 (13)	−0.0071 (13)	−0.0135 (13)
C16	0.046 (2)	0.064 (2)	0.0444 (19)	0.0060 (17)	−0.0116 (16)	−0.0131 (17)
C17	0.0387 (18)	0.050 (2)	0.0456 (18)	0.0053 (15)	−0.0141 (15)	−0.0155 (16)
C18	0.051 (2)	0.056 (2)	0.054 (2)	0.0006 (17)	−0.0130 (17)	−0.0162 (17)
C19	0.056 (2)	0.069 (3)	0.052 (2)	−0.005 (2)	−0.0029 (18)	−0.0267 (19)
C20	0.045 (2)	0.077 (3)	0.070 (3)	0.012 (2)	−0.0156 (19)	−0.040 (2)
C21	0.057 (2)	0.071 (3)	0.063 (2)	0.019 (2)	−0.027 (2)	−0.025 (2)
C22	0.047 (2)	0.052 (2)	0.0402 (18)	0.0013 (17)	−0.0081 (16)	−0.0086 (16)
C23	0.0441 (18)	0.0427 (19)	0.0399 (17)	0.0017 (15)	−0.0081 (15)	−0.0126 (15)
C24	0.049 (2)	0.059 (2)	0.051 (2)	0.0140 (17)	−0.0038 (17)	−0.0065 (17)
C25	0.0372 (18)	0.045 (2)	0.0478 (19)	0.0061 (15)	−0.0070 (15)	−0.0073 (16)
C26	0.064 (2)	0.065 (2)	0.051 (2)	−0.010 (2)	−0.0216 (18)	−0.0108 (19)
C27	0.073 (3)	0.062 (3)	0.057 (2)	−0.020 (2)	−0.014 (2)	−0.002 (2)
C28	0.064 (2)	0.075 (3)	0.048 (2)	0.002 (2)	−0.0136 (19)	−0.004 (2)
C29	0.064 (2)	0.093 (3)	0.060 (2)	0.000 (2)	−0.026 (2)	−0.024 (2)
C30	0.044 (2)	0.060 (2)	0.077 (3)	−0.0071 (18)	−0.0144 (19)	−0.019 (2)
Cl1	0.0795 (7)	0.0724 (7)	0.0449 (5)	0.0055 (5)	−0.0195 (5)	−0.0065 (4)
S1	0.0544 (5)	0.0630 (6)	0.0368 (5)	0.0098 (4)	−0.0037 (4)	−0.0044 (4)
S2	0.0575 (6)	0.0645 (6)	0.0414 (5)	0.0168 (5)	−0.0038 (4)	−0.0092 (4)
N1	0.0471 (16)	0.0566 (18)	0.0324 (14)	0.0087 (14)	−0.0064 (12)	−0.0090 (13)
N2	0.0382 (15)	0.0541 (17)	0.0400 (15)	0.0016 (13)	−0.0064 (12)	−0.0133 (13)
C1	0.050 (2)	0.053 (2)	0.0430 (19)	0.0028 (17)	−0.0150 (16)	−0.0158 (16)
C2	0.0414 (18)	0.048 (2)	0.0406 (18)	−0.0013 (15)	−0.0083 (15)	−0.0144 (15)
C3	0.056 (2)	0.061 (2)	0.045 (2)	0.0045 (18)	−0.0118 (17)	−0.0111 (17)
C4	0.058 (2)	0.092 (3)	0.049 (2)	0.005 (2)	−0.0011 (19)	−0.019 (2)
C5	0.050 (2)	0.108 (4)	0.075 (3)	0.021 (2)	−0.010 (2)	−0.038 (3)
C6	0.057 (2)	0.078 (3)	0.065 (3)	0.019 (2)	−0.025 (2)	−0.025 (2)
C7	0.0427 (18)	0.050 (2)	0.0369 (17)	0.0004 (16)	−0.0086 (15)	−0.0086 (15)
C8	0.0452 (18)	0.0440 (19)	0.0380 (17)	0.0020 (15)	−0.0105 (15)	−0.0095 (15)
C9	0.059 (2)	0.061 (2)	0.044 (2)	0.0099 (18)	−0.0101 (17)	−0.0044 (17)
C10	0.053 (2)	0.044 (2)	0.0389 (17)	0.0089 (16)	−0.0134 (16)	−0.0101 (15)
C11	0.065 (2)	0.049 (2)	0.053 (2)	−0.0041 (18)	−0.0205 (18)	−0.0095 (17)
C12	0.076 (3)	0.070 (3)	0.047 (2)	0.009 (2)	−0.026 (2)	−0.0189 (19)
C13	0.077 (3)	0.060 (2)	0.0364 (19)	0.010 (2)	−0.0092 (19)	−0.0085 (17)
C14	0.066 (2)	0.065 (3)	0.055 (2)	−0.011 (2)	−0.007 (2)	−0.011 (2)
C15	0.066 (2)	0.069 (3)	0.050 (2)	−0.003 (2)	−0.0174 (19)	−0.0199 (19)

### Geometric parameters (Å, °)

**Table:**

Cl2—C16	1.742 (3)	Cl1—C1	1.738 (3)
S3—C23	1.750 (3)	S1—C8	1.751 (3)
S3—C24	1.815 (3)	S1—C9	1.810 (3)
S4—C23	1.662 (3)	S2—C8	1.657 (3)
N3—C23	1.337 (4)	N1—C8	1.334 (4)
N3—N4	1.376 (3)	N1—N2	1.375 (3)
N3—H3A	0.8600	N1—H1	0.8600
N4—C22	1.271 (4)	N2—C7	1.279 (4)
C16—C17	1.384 (4)	C1—C6	1.377 (5)
C16—C21	1.384 (5)	C1—C2	1.390 (4)
C17—C18	1.399 (4)	C2—C3	1.398 (4)
C17—C22	1.458 (4)	C2—C7	1.455 (4)
C18—C19	1.380 (5)	C3—C4	1.376 (5)
C18—H18	0.9300	C3—H3	0.9300
C19—C20	1.375 (5)	C4—C5	1.371 (5)
C19—H19	0.9300	C4—H4	0.9300
C20—C21	1.358 (5)	C5—C6	1.366 (5)
C20—H20	0.9300	C5—H5	0.9300
C21—H21	0.9300	C6—H6	0.9300
C22—H22	0.9300	C7—H7	0.9300
C24—C25	1.503 (4)	C9—C10	1.512 (4)
C24—H24A	0.9700	C9—H9A	0.9700
C24—H24B	0.9700	C9—H9B	0.9700
C25—C26	1.373 (4)	C10—C15	1.374 (4)
C25—C30	1.379 (5)	C10—C11	1.378 (4)
C26—C27	1.377 (5)	C11—C12	1.384 (5)
C26—H26	0.9300	C11—H11	0.9300
C27—C28	1.366 (5)	C12—C13	1.364 (5)
C27—H27	0.9300	C12—H12	0.9300
C28—C29	1.355 (5)	C13—C14	1.377 (5)
C28—H28	0.9300	C13—H13	0.9300
C29—C30	1.381 (5)	C14—C15	1.382 (5)
C29—H29	0.9300	C14—H14	0.9300
C30—H30	0.9300	C15—H15	0.9300
			
C23—S3—C24	101.41 (15)	C8—S1—C9	101.13 (15)
C23—N3—N4	121.2 (2)	C8—N1—N2	121.0 (2)
C23—N3—H3A	119.4	C8—N1—H1	119.5
N4—N3—H3A	119.4	N2—N1—H1	119.5
C22—N4—N3	115.6 (3)	C7—N2—N1	115.5 (3)
C17—C16—C21	122.5 (3)	C6—C1—C2	121.8 (3)
C17—C16—Cl2	120.3 (3)	C6—C1—Cl1	117.5 (3)
C21—C16—Cl2	117.3 (3)	C2—C1—Cl1	120.7 (3)
C16—C17—C18	116.9 (3)	C1—C2—C3	117.1 (3)
C16—C17—C22	122.2 (3)	C1—C2—C7	121.8 (3)
C18—C17—C22	120.9 (3)	C3—C2—C7	121.0 (3)
C19—C18—C17	120.8 (3)	C4—C3—C2	121.1 (3)
C19—C18—H18	119.6	C4—C3—H3	119.5
C17—C18—H18	119.6	C2—C3—H3	119.5
C20—C19—C18	120.3 (3)	C5—C4—C3	119.9 (4)
C20—C19—H19	119.9	C5—C4—H4	120.1
C18—C19—H19	119.9	C3—C4—H4	120.1
C21—C20—C19	120.5 (3)	C6—C5—C4	120.6 (4)
C21—C20—H20	119.8	C6—C5—H5	119.7
C19—C20—H20	119.8	C4—C5—H5	119.7
C20—C21—C16	119.2 (3)	C5—C6—C1	119.5 (4)
C20—C21—H21	120.4	C5—C6—H6	120.3
C16—C21—H21	120.4	C1—C6—H6	120.3
N4—C22—C17	121.6 (3)	N2—C7—C2	120.9 (3)
N4—C22—H22	119.2	N2—C7—H7	119.6
C17—C22—H22	119.2	C2—C7—H7	119.6
N3—C23—S4	121.1 (2)	N1—C8—S2	121.6 (2)
N3—C23—S3	114.0 (2)	N1—C8—S1	113.4 (2)
S4—C23—S3	124.88 (19)	S2—C8—S1	125.03 (19)
C25—C24—S3	108.6 (2)	C10—C9—S1	108.5 (2)
C25—C24—H24A	110.0	C10—C9—H9A	110.0
S3—C24—H24A	110.0	S1—C9—H9A	110.0
C25—C24—H24B	110.0	C10—C9—H9B	110.0
S3—C24—H24B	110.0	S1—C9—H9B	110.0
H24A—C24—H24B	108.4	H9A—C9—H9B	108.4
C26—C25—C30	117.7 (3)	C15—C10—C11	118.4 (3)
C26—C25—C24	121.6 (3)	C15—C10—C9	121.5 (3)
C30—C25—C24	120.8 (3)	C11—C10—C9	120.0 (3)
C25—C26—C27	121.3 (3)	C10—C11—C12	120.4 (3)
C25—C26—H26	119.4	C10—C11—H11	119.8
C27—C26—H26	119.4	C12—C11—H11	119.8
C28—C27—C26	120.1 (3)	C13—C12—C11	120.6 (3)
C28—C27—H27	119.9	C13—C12—H12	119.7
C26—C27—H27	119.9	C11—C12—H12	119.7
C29—C28—C27	119.6 (3)	C12—C13—C14	119.6 (3)
C29—C28—H28	120.2	C12—C13—H13	120.2
C27—C28—H28	120.2	C14—C13—H13	120.2
C28—C29—C30	120.5 (4)	C13—C14—C15	119.6 (3)
C28—C29—H29	119.8	C13—C14—H14	120.2
C30—C29—H29	119.8	C15—C14—H14	120.2
C25—C30—C29	120.9 (3)	C10—C15—C14	121.3 (3)
C25—C30—H30	119.6	C10—C15—H15	119.3
C29—C30—H30	119.6	C14—C15—H15	119.3
			
C23—N3—N4—C22	−174.7 (3)	C8—N1—N2—C7	178.3 (3)
C21—C16—C17—C18	−0.1 (5)	C6—C1—C2—C3	−0.5 (5)
Cl2—C16—C17—C18	179.5 (3)	Cl1—C1—C2—C3	−179.9 (3)
C21—C16—C17—C22	−178.8 (3)	C6—C1—C2—C7	178.3 (3)
Cl2—C16—C17—C22	0.8 (5)	Cl1—C1—C2—C7	−1.0 (4)
C16—C17—C18—C19	0.7 (5)	C1—C2—C3—C4	−0.3 (5)
C22—C17—C18—C19	179.4 (3)	C7—C2—C3—C4	−179.2 (3)
C17—C18—C19—C20	−0.9 (5)	C2—C3—C4—C5	0.8 (6)
C18—C19—C20—C21	0.5 (6)	C3—C4—C5—C6	−0.6 (6)
C19—C20—C21—C16	0.1 (6)	C4—C5—C6—C1	−0.2 (6)
C17—C16—C21—C20	−0.3 (6)	C2—C1—C6—C5	0.8 (6)
Cl2—C16—C21—C20	−179.9 (3)	Cl1—C1—C6—C5	−179.8 (3)
N3—N4—C22—C17	179.8 (3)	N1—N2—C7—C2	179.0 (3)
C16—C17—C22—N4	−176.4 (3)	C1—C2—C7—N2	−174.0 (3)
C18—C17—C22—N4	5.0 (5)	C3—C2—C7—N2	4.8 (5)
N4—N3—C23—S4	176.9 (2)	N2—N1—C8—S2	−179.3 (2)
N4—N3—C23—S3	−2.0 (4)	N2—N1—C8—S1	0.3 (4)
C24—S3—C23—N3	−179.2 (3)	C9—S1—C8—N1	177.0 (2)
C24—S3—C23—S4	1.8 (3)	C9—S1—C8—S2	−3.5 (3)
C23—S3—C24—C25	−174.0 (2)	C8—S1—C9—C10	−174.1 (2)
S3—C24—C25—C26	−80.4 (4)	S1—C9—C10—C15	71.2 (4)
S3—C24—C25—C30	99.9 (3)	S1—C9—C10—C11	−110.1 (3)
C30—C25—C26—C27	0.4 (5)	C15—C10—C11—C12	0.9 (5)
C24—C25—C26—C27	−179.4 (3)	C9—C10—C11—C12	−177.8 (3)
C25—C26—C27—C28	0.2 (6)	C10—C11—C12—C13	−0.6 (6)
C26—C27—C28—C29	−0.1 (6)	C11—C12—C13—C14	0.2 (6)
C27—C28—C29—C30	−0.5 (6)	C12—C13—C14—C15	−0.1 (6)
C26—C25—C30—C29	−0.9 (5)	C11—C10—C15—C14	−0.9 (6)
C24—C25—C30—C29	178.8 (3)	C9—C10—C15—C14	177.8 (3)
C28—C29—C30—C25	1.0 (6)	C13—C14—C15—C10	0.5 (6)

### Hydrogen-bond geometry (Å, °)

**Table:**

*D*—H···*A*	*D*—H	H···*A*	*D*···*A*	*D*—H···*A*
N1—H1···S4	0.86	2.56	3.405 (3)	166
N3—H3A···S2	0.86	2.60	3.451 (3)	169
